# The *in vitro* effect of new combinations of carbapenem–β-lactamase inhibitors for *Mycobacterium abscessus*

**DOI:** 10.1128/aac.00528-23

**Published:** 2023-09-06

**Authors:** Gabrielle Fröberg, Ayan Ahmed, Erja Chryssanthou, Lina Davies Forsman

**Affiliations:** 1 Department of Clinical Microbiology, Karolinska University Hospital, Stockholm, Sweden; 2 Department of Medicine, Division of Infectious Diseases, Karolinska Institutet, Solna, Sweden; 3 Department of Infectious Diseases, Karolinska University Hospital, Stockholm, Sweden; University of Fribourg, Fribourg, Switzerland

**Keywords:** new treatment alternatives, minimum inhibitory concentrations, β-lactam antibiotics, β-lactamase inhibitors, difficult to treat NTMs, imipenem-relebactam, tebipenem-avibactam

## Abstract

As new treatment alternatives for *Mycobacterium abscessus* complex (MABC) are urgently needed, we determined the minimum inhibitory concentrations (MICs) for novel carbapenem combinations, including imipenem-relebactam and tebipenem-avibactam against 98 MABC isolates by broth microdilution. The MIC50 was reduced from 16 to 8 mg/L by adding relebactam to imipenem, while the addition of avibactam to tebipenem showed a more pronounced reduction from 256 to 16 mg/L, representing a promising non-toxic, oral treatment option for further exploration.

## INTRODUCTION

Non-tuberculous mycobacteria (NTM) are increasing globally, of which *Mycobacterium abscessus* complex (MABC) is particularly difficult to treat. Despite long treatment durations with multiple drugs, treatment success in Europe is only 30%–50% (1) and worse for subspecies *abscessus* (33%) compared to subspecies *massiliense* (56%) (2).

Due to intrinsic and acquired resistance of MABC to first-line drugs such as macrolides (*erm* and *rrl* genes) and aminoglycosides (*rrs* gene), few treatment alternatives exist (3), and repurposed drugs are currently highlighted for possible treatment options (4). MABC is highly resistant to most β-lactam antibiotics, and the current guidelines include only imipenem and cefoxitin (5). The major challenge to the use of β-lactam antibiotics against MABC is the β-lactamase *BlaMab*, requiring the addition of a β-lactamase inhibitor (BLI) (6). The novel intravenous combination of imipenem-relebactam is approved for resistant Gram-negative infections (7), and the oral carbapenem tebipenem was recently shown effective against complicated urinary tract infections (8). Relebactam is structurally related to avibactam, which has been shown to inhibit *BlaMab* (7).

We have previously shown an association between rough colony morphology and poor treatment outcome of MABC infection (9), and we hypothesized that this could be due to a possible association with drug resistance. Therefore, we have determined the *in vitro* effect of imipenem-relebactam and tebipenem-avibactam against MABC isolates, exploring the potential effect of MABC subspecies, *erm* and colony morphology on minimum inhibitory concentration (MIC) levels.

Frozen MABC clinical isolates from Sweden collected during 2009–2021 (*n* = 92) and six isolates from an external quality panel (Instand, Germany) 2020–2021 were thawed and cultured on Löwenstein-Jensen medium at 30°C. Data were available regarding cording (by fluorescent microscopy), MABC subspecies, i.e., *abscessus*, *massiliense*, and *bolletii*, as well as *erm*, *rrl*, *and rrs* genotype (Genotype NTM-DR, HAIN LifeSciences, Germany) (10). MABC colony morphology, i.e., smooth, rough, or mixed, was determined by light microscopy as previously described (9).

MICs of carbapenems alone and in combination with BLIs were determined by broth microdilution (BMD) in cation-adjusted BBL Mueller-Hinton II broth (MH). Imipenem and avibactam (MedChemExpress, USA), tebipenem (Spero Therapeutics, USA), and relebactam (Merck, Germany) compounds were solved in H2O, phosphate buffer, or dimethyl sulfoxide (DMSO) according to manufacturer’s instructions, and aliquoted stock solutions were frozen in −80°C. On the day of testing, stock solutions were diluted in MH broth, and 100 µL was dispersed into 96 well plates, where carbapenems were distributed by stepwise 1:2 dilutions from 256 to 0.5 mg/L, and BLIs were distributed at a fix final test concentration of 4 mg/L, informed by previous studies (11 – 14). MABC colonies from growing cultures were suspended in MH broth to ca. 5 × 10^5^ CFU/mL, and 100 µL was distributed in all wells. MIC was defined as the lowest drug concentration without visible bacterial growth after 3–5 days of incubation at 30°C. Reference strains *Mycobacterium peregrinum* ATCC 700686 and *M. abscessus* ATCC 19977 were included as quality controls (QC). Mann-Whitney or Kruskal-Wallis one-way ANOVA was used to compare MIC values between groups. *P*-value <0.05 was considered statistically significant.

**Fig 1:**
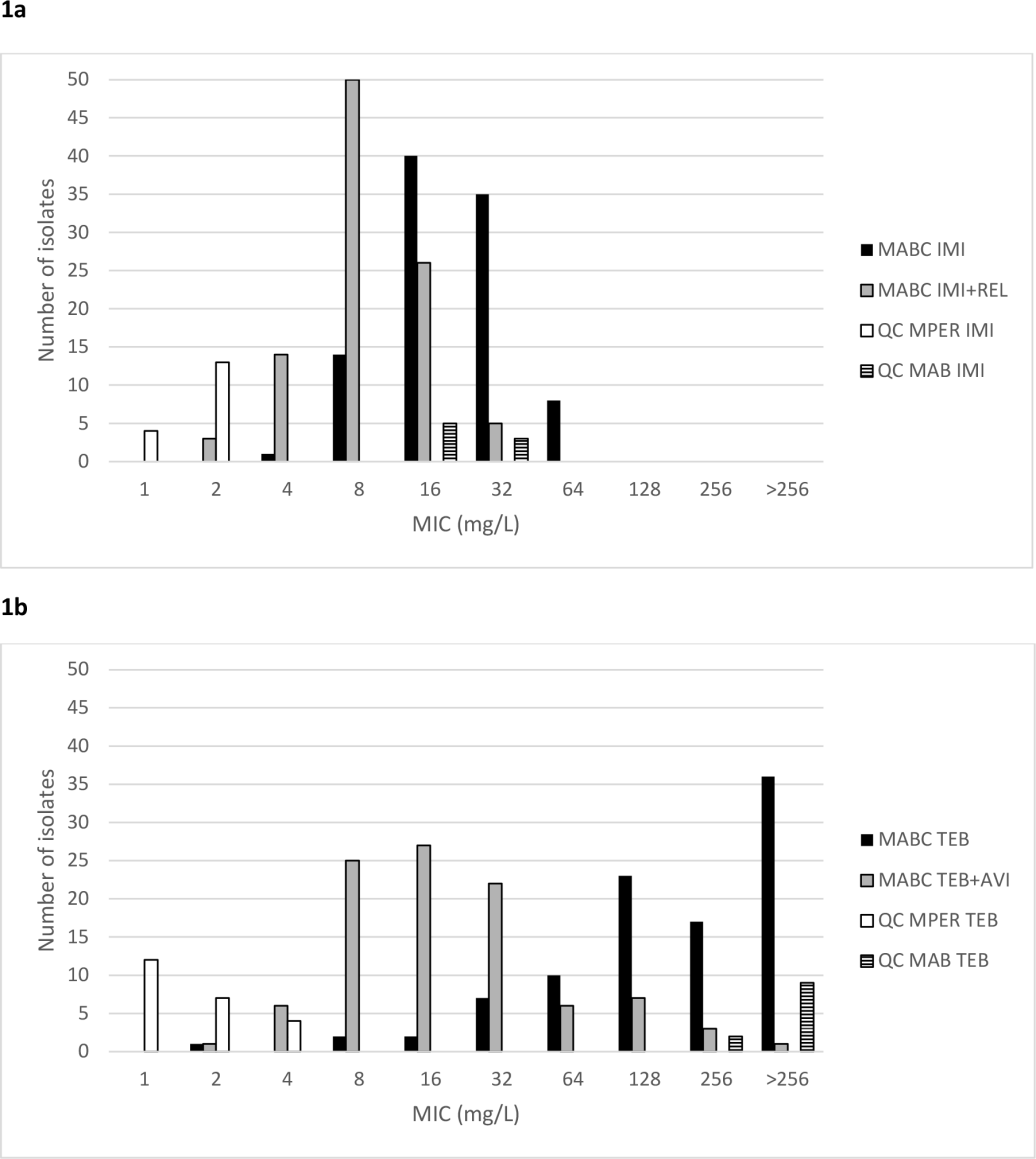
MIC distribution of (a) imipenem (IMI) ± relebactam (REL) and (b) tebipenem (TEB) ± avibactam (AVI). QC MPER, quality control *M. peregrinum* ATCC 700686; QC MAB, quality control *M. abscessus* ATCC 19977. The majority of the 98 isolates were clinical, whereas 6 isolates were part of an external control panel (Instand, Germany).

Characteristics of MABC isolates are summarized in Table 1. MIC including 50% of the isolates (MIC50) was reduced one dilution step from 16 to 8 mg/L by adding relebactam to imipenem, while the addition of avibactam to tebipenem showed a pronounced reduction of the MIC50 from 256 to 16 mg/L (Fig. 1a and b) (*P* < 0.001). For QC *M. peregrinum and M. abscessus*, median MICs for imipenem were 2 and 16 mg/L, while for tebipenem, median MICs were 1 and >256 mg/L, respectively, with ranges within ±1 dilution step.

**TABLE 1:** Characteristics of MABC isolates^*a*^

MAB subspecies	Total (*n*)	Cording *n* (%)	Morphology *n* (%)			*erm*(41) *n* (%)	rrl *n* (%)	rrs *n* (%)
		Detected	Smooth	Rough	Mixed	28T	Mutation	Mutation
*M. abscessus*	53	19 (36%)	22 (42%)	22 (42%)	9 (17%)	40 (75%)	0	0
*M. massiliense*	26	6 (23%)	17 (65%)	6 (23%)	3 (12%)	Inactive	1^*b*^ (4%)	0
*M. bolletii*	19	2 (11%)	14 (74%)	5 (26%)	0	19 (100%)	0	0

^*a*^ Number of isolates and percentage of the total number of respective subspecies (within brackets) with regards to the formation of cording, colony morphology, and resistant genotype of erm(41), rrl, or rrs. Mixed morphology was defined as the occurrence of smooth and rough colonies in the same isolate.

^*b*^ rrl A2058G/T.

For imipenem alone, similar MIC levels were found in a large study including over a thousand of MABC isolates as determined by BMD in MH (15). In a smaller study including 28 MABC isolates, the addition of relebactam to imipenem resulted in the same reduction in modal MIC from 16 to 8 mg/L as in our study (BMD 7H9) (16), a difference that may, however, be explained by the innate variability of any MIC determination (17).

Our results regarding tebipenem are corroborated by previously published studies. Kaushik et al. identified avibactam as the most effective BLI in lowering the MABC MICs of tebipenem, in a study including eight different carbapenems combined with avibactam, sulbactam, or tazobactam, respectively (BMD 7H9). The modal tebipenem MIC of 256 mg/L was reduced to 8 mg/L (range 4–16 mg/L) when combined with avibactam (*n* = 28) (11). Avibactam has been shown to inhibit *BlaMab* more rapidly than relebactam in a hydrolysis kinetics study (18). Only a modest reduction has been seen by the addition of clavulanic acid 5 mg/L for *M. abscessus* ATCC 19,977, as compared with the effect on *BlaC* expressed by *Mycobacterium tuberculosis* (19, 20).

As for the high MIC values of tebipenem tested singly, similar MIC50s of 256 to >256 mg/L have been previously reported (11, 16, 21). In another study including 19 clinical MABC isolates, the MIC of the majority of isolates was above the upper test concentration of 256 mg/L, with only two isolates with MICs below 256 mg/L (22).

Regarding colony morphology, there was a tendency of lower tebipenem MICs for rough compared to smooth morphology (MIC50 128 vs 256 mg/L); however, this was not statistically significant (*P* = 0.064). Otherwise, there were no significant differences in MIC50s for any of the carbapenem alone or in combination with BLI with regard to (a) MABC subspecies, (b) *erm* genotype, (c) formation of cording, or (d) morphology (see https://doi.org/10.6084/m9.figshare.23589786.v1), which is in line with previous research (23 – 28). From our data, a rough colony morphology does not appear to be a major mechanism of resistance to carbapenems (9).

*In vivo* studies and clinical breakpoints for imipenem-relebactam or tebipenem-avibactam are lacking for MABC. The MIC breakpoints of imipenem are defined by Clinial & Laboratory Standards Institute (CLSI) as ≤4 mg/L for susceptible, 8–16 mg/L for intermediate, and ≥32 mg/L for resistant (29). Based on our results, the combination of imipenem-relebactam means that the majority of MABC isolates would fall into the intermediate category (MIC50 8 mg/L) and as such, still two dilution steps above the non-species related PK/PD breakpoint (MIC ≤2 mg/L) as suggested by the European Committee on Antimicrobial Susceptibility Testing (EUCAST), indicating that combination therapies are still needed to enhance activity.

As for tebipenem, the mean *C*_max_ values for tebipenem was 15.1 mg/L following 600 mg of a prodrug of tebipenem thrice daily for 2 wk in 30 healthy volunteers (8). In a mural model, the bacterial growth increased as soon as the free plasma concentrations of tebipenem were less than the MIC of the isolate (30). An oral prodrug of avibactam (AVI-006) is included in phase I clinical trial (ClinicalTrials.gov NCT03931876). Promising *in vivo* activity has been shown in a mouse model by this combination, claiming satisfying PK/PD data and likely bactericidal effect of MABC pulmonary infection (13).

In conclusion, the role of the all-oral combination tebipenem-avibactam for MABC may deserve further exploration.

## References

[1] Jarand J , Levin A , Zhang L , Huitt G , Mitchell JD , Daley CL . 2011. Clinical and microbiologic outcomes in patients receiving treatment for Mycobacterium abscessus pulmonary disease. Clin Infect Dis 52:565–571. doi:10.1093/cid/ciq237 21292659

[2] Kwak N , Dalcolmo MP , Daley CL , Eather G , Gayoso R , Hasegawa N , Jhun BW , Koh WJ , Namkoong H , Park J , Thomson R , van Ingen J , Zweijpfenning SMH , Yim JJ . 2019. Mycobacterium abscessus pulmonary disease: individual patient data meta-analysis. Eur Respir J 54. doi:10.1183/13993003.01991-2018 30880280

[3] Nessar R , Cambau E , Reyrat JM , Murray A , Gicquel B . 2012. Mycobacterium abscessus: a new antibiotic nightmare. J Antimicrob Chemother 67:810–818. doi:10.1093/jac/dkr578 22290346

[4] Alffenaar J-W , Sintchenko V , Marais BJ . 2019. Acquired drug resistance: recognizing potential of repurposed drugs. Clin Infect Dis 69:2038–2039. doi:10.1093/cid/ciz334 31125392

[5] Daley CL , Iaccarino JM , Lange C , Cambau E , Wallace RJ , Andrejak C , Böttger EC , Brozek J , Griffith DE , Guglielmetti L , Huitt GA , Knight SL , Leitman P , Marras TK , Olivier KN , Santin M , Stout JE , Tortoli E , van Ingen J , Wagner D , Winthrop KL . 2020. Treatment of nontuberculous mycobacterial pulmonary disease: an official ATS/ERS/ESCMID/IDSA clinical practice guideline. Clin Infect Dis 71:905–913. doi:10.1093/cid/ciaa1125 32797222 PMC7768745

[6] Story-Roller E , Maggioncalda EC , Cohen KA , Lamichhane G . 2018. Mycobacterium abscessus and β-lactams: emerging insights and potential opportunities. Front Microbiol 9:2273. doi:10.3389/fmicb.2018.02273 30319581 PMC6167491

[7] Zhanel GG , Lawrence CK , Adam H , Schweizer F , Zelenitsky S , Zhanel M , Lagacé-Wiens PRS , Walkty A , Denisuik A , Golden A , Gin AS , Hoban DJ , Lynch JP , Karlowsky JA . 2018. Imipenem-relebactam and meropenem-vaborbactam: two novel carbapenem-β-lactamase inhibitor combinations. Drugs 78:65–98. doi:10.1007/s40265-017-0851-9 29230684

[8] Eckburg PB , Muir L , Critchley IA , Walpole S , Kwak H , Phelan A-M , Moore G , Jain A , Keutzer T , Dane A , Melnick D , Talley AK . 2022. Oral tebipenem pivoxil hydrobromide in complicated urinary tract infection. N Engl J Med 386:1327–1338. doi:10.1056/NEJMoa2105462 35388666

[9] Hedin W , Fröberg G , Fredman K , Chryssanthou E , Selmeryd I , Gillman A , Orsini L , Runold M , Jönsson B , Schön T , Davies Forsman L . 2023. A rough colony morphology of Mycobacterium abscessus is associated with cavitary pulmonary disease and poor clinical outcome. J Infect Dis 227:820–827. doi:10.1093/infdis/jiad007 36637124 PMC10043986

[10] BRUKER . n.d. Available from: https://www.hain-lifescience.de/en/products/microbiology/mycobacteria/ntm/genotype-ntm-dr.html

[11] Kaushik A , Gupta C , Fisher S , Story-Roller E , Galanis C , Parrish N , Lamichhane G . 2017. Combinations of avibactam and carbapenems exhibit enhanced potencies against drug-resistant Mycobacterium abscessus. Future Microbiol 12:473–480. doi:10.2217/fmb-2016-0234 28326811 PMC5618940

[12] Horita Y , Maeda S , Kazumi Y , Doi N . 2014. In vitro susceptibility of Mycobacterium tuberculosis isolates to an oral carbapenem alone or in combination with β-lactamase inhibitors. Antimicrob Agents Chemother 58:7010–7014. doi:10.1128/AAC.03539-14 25224000 PMC4249422

[13] Negatu DA , González Del Río R , Cacho-Izquierdo M , Barros-Aguirre D , Lelievre J , Rullas J , Casado P , Ganapathy US , Zimmerman MD , Gengenbacher M , Dartois V , Dick T . 2023. Activity of oral tebipenem-avibactam in a mouse model of Mycobacterium abscessus lung infection. Antimicrob Agents Chemother 67:e0145922. doi:10.1128/aac.01459-22 36688684 PMC9933631

[14] Negatu DA , Zimmerman MD , Dartois V , Dick T . 2022. Strongly bactericidal all-oral β-lactam combinations for the treatment of Mycobacterium abscessus lung disease. Antimicrob Agents Chemother 66:e0079022. doi:10.1128/aac.00790-22 36047786 PMC9487536

[15] Fröberg G , Maurer FP , Chryssanthou E , Fernström L , Benmansour H , Boarbi S , Mengshoel AT , Keller PM , Viveiros M , Machado D , Fitzgibbon MM , Mok S , Werngren J , Cirillo DM , Alcaide F , Hyyryläinen H-L , Aubry A , Andres S , Nadarajan D , Svensson E , Turnidge J , Giske CG , Kahlmeter G , Cambau E , van Ingen J , Schön T , EUCAST AMST and ESCMYC study groups . 2023. Towards clinical breakpoints for non-tuberculous mycobacteria - determination of epidemiological cut off values for the mycobacterium avium complex and Mycobacterium abscessus using broth microdilution. Clin Microbiol Infect 29:758–764. doi:10.1016/j.cmi.2023.02.007 36813087

[16] Kaushik A , Ammerman NC , Lee J , Martins O , Kreiswirth BN , Lamichhane G , Parrish NM , Nuermberger EL . 2019. In vitro activity of the new β-lactamase inhibitors relebactam and vaborbactam in combination with β-lactams against Mycobacterium abscessus complex clinical isolates. Antimicrob Agents Chemother 63:e02623-18. doi:10.1128/AAC.02623-18 30642943 PMC6395916

[17] Mouton JW , Meletiadis J , Voss A , Turnidge J . 2018. Variation of MIC measurements: the contribution of strain and laboratory variability to measurement precision. J Antimicrob Chemother 73:2374–2379. doi:10.1093/jac/dky232 30137390

[18] Dousa KM , Kurz SG , Taracila MA , Bonfield T , Bethel CR , Barnes MD , Selvaraju S , Abdelhamed AM , Kreiswirth BN , Boom WH , Kasperbauer SH , Daley CL , Bonomo RA . 2020. Insights into the l,d-transpeptidases and d,d-carboxypeptidase of Mycobacterium abscessus: ceftaroline, imipenem, and novel diazabicyclooctane inhibitors. Antimicrob Agents Chemother 64:e00098-20. doi:10.1128/AAC.00098-20 32393499 PMC7526840

[19] Kaushik A , Makkar N , Pandey P , Parrish N , Singh U , Lamichhane G . 2015. Carbapenems and rifampin exhibit synergy against Mycobacterium tuberculosis and Mycobacterium abscessus. Antimicrob Agents Chemother 59:6561–6567. doi:10.1128/AAC.01158-15 26259792 PMC4576034

[20] Dubée V , Bernut A , Cortes M , Lesne T , Dorchene D , Lefebvre A-L , Hugonnet J-E , Gutmann L , Mainardi J-L , Herrmann J-L , Gaillard J-L , Kremer L , Arthur M . 2015. β-lactamase inhibition by avibactam in Mycobacterium abscessus. J Antimicrob Chemother 70:1051–1058. doi:10.1093/jac/dku510 25525201

[21] Misawa K , Nishimura T , Kashimura S , Enoki Y , Taguchi K , Uno S , Uwamino Y , Matsumoto K , Hasegawa N . 2022. In vitro effects of diazabicyclooctane β-lactamase inhibitors relebactam and nacubactam against three subspecies of Mycobacterium abscessus complex. Int J Antimicrob Agents 60:106669. doi:10.1016/j.ijantimicag.2022.106669 36064079

[22] Gumbo T , Cirrincione K , Srivastava S . 2020. Repurposing drugs for treatment of Mycobacterium abscessus: a view to a kill. J Antimicrob Chemother 75:1212–1217. doi:10.1093/jac/dkz523 32016429

[23] Fujiwara K , Uesugi F , Furuuchi K , Tanaka Y , Yoshiyama T , Saotome M , Ohta K , Mitarai S , Morimoto K . 2021. Minimum inhibitory concentrations before and after antibacterial treatment in patients with Mycobacterium abscessus pulmonary disease. Microbiol Spectr 9:e0192821. doi:10.1128/Spectrum.01928-21 34878300 PMC8653840

[24] Guo Y , Cao X , Yu J , Zhan Q , Yang J , Wu X , Wan B , Liu Y , Yu F . 2020. Antimicrobial susceptibility of Mycobacterium abscessus complex clinical isolates from a Chinese tertiary hospital. Infect Drug Resist 13:2001–2010. doi:10.2147/IDR.S252485 32617011 PMC7326206

[25] Lavollay M , Dubée V , Heym B , Herrmann J-L , Gaillard J-L , Gutmann L , Arthur M , Mainardi J-L . 2014. In vitro activity of cefoxitin and imipenem against Mycobacterium abscessus complex. Clin Microbiol Infect 20:297–300. doi:10.1111/1469-0691.12405 24112243

[26] Li G , Pang H , Guo Q , Huang M , Tan Y , Li C , Wei J , Xia Y , Jiang Y , Zhao X , Liu H , Zhao L-L , Liu Z , Xu D , Wan K . 2017. Antimicrobial susceptibility and MIC distribution of 41 drugs against clinical isolates from China and reference strains of nontuberculous mycobacteria. Int J Antimicrob Agents 49:364–374. doi:10.1016/j.ijantimicag.2016.10.024 28131606

[27] Story-Roller E , Galanis C , Lamichhane G . 2021. β-lactam combinations that exhibit synergy against Mycobacteroides abscessus clinical isolates. Antimicrob Agents Chemother 65:e02545-20. doi:10.1128/AAC.02545-20 33361310 PMC8097488

[28] Kaushik A , Ammerman NC , Parrish NM , Nuermberger EL . 2019. New β-lactamase inhibitors nacubactam and zidebactam improve the in vitro activity of β-lactam antibiotics against Mycobacterium abscessus complex clinical isolates. Antimicrob Agents Chemother 63:e00733-19. doi:10.1128/AAC.00733-19 31209013 PMC6709484

[29] CLSI . 2020. Susceptibility testing of mycobacteria, nocardia spp., and other aerobic actinomycetes. 3rd ed. Standard M24. 31339680

[30] McEntee L , Johnson A , Farrington N , Unsworth J , Dane A , Jain A , Cotroneo N , Critchley I , Melnick D , Parr T , Ambrose PG , Das S , Hope W . 2019. Pharmacodynamics of tebipenem: new options for oral treatment of multidrug-resistant gram-negative infections. Antimicrob Agents Chemother 63:e00603-19. doi:10.1128/AAC.00603-19 31109982 PMC6658774

